# Functional, imaging, and respiratory evaluation (FIRE) of patients post-hospitalization for COVID-19: protocol for a pilot observational study

**DOI:** 10.1186/s40814-022-01151-8

**Published:** 2022-09-19

**Authors:** Kirby P. Mayer, Jessica A. Palakshappa, Ithan Daniel Peltan, James S. Andrew, Stephanie J. Gundel, Nancy J. Ringwood, Jeffrey Mckeehan, Aluko A. Hope, Angela J. Rogers, Michelle Biehl, Douglas L. Hayden, Ellen Caldwell, Omar Mehkri, David J. Lynch, Ellen L. Burham, Catherine L. Hough, Sarah E. Jolley, for the NHLBI PETAL Network

**Affiliations:** 1 Division of Pulmonary and Critical Care Medicine, University of Colorado Anschutz Medical Campus, 12700 East 19th Avenue, Aurora, CO 80045 USA; 2 Department of Physical Therapy, University of Kentucky, Lexington, USA; 3 Section of Pulmonary, Critical Care, Allergy, and Immunologic Diseases, Wake Forest University School of Medicine, Winston-Salem, USA; 4 Intermountain Healthcare, Salt Lake City, USA; 5 Division of Rheumatology, University of Washington, Seattle, USA; 6 University of Washington, Seattle, USA; 7 Massachusetts General Hospital, Boston, USA; 8 Division of Pulmonary and Critical Care Medicine, University of Colorado Anschutz Medical Campus, Aurora, CO USA; 9 Pulmonary and Critical Care Medicine, Oregon Health and Sciences University, Portland, USA; 10 Pulmonary and Critical Care Medicine, Stanford University, Stanford, USA; 11 Critical Care Medicine, Cleveland Clinic, Cleveland, USA; 12 Division of Pulmonary and Critical Care, University of Washington, Seattle, USA; 13 Department of Critical Care, Respiratory Institute, Cleveland Clinic, Cleveland, USA; 14 Department of Radiology, National Jewish Health, Denver, USA; 15 University of Colorado Anschutz Medical Campus, Aurora, CO USA

**Keywords:** COVID19, ARDS, Epidemiology

## Abstract

**Introduction:**

We describe a protocol for FIRE CORAL, an observational cohort study that examines the recovery from COVID-19 disease following acute hospitalization with an emphasis on functional, imaging, and respiratory evaluation.

**Methods and analysis:**

FIRE CORAL is a multicenter prospective cohort study of participants recovering from COVID-19 disease with in-person follow-up for functional and pulmonary phenotyping conducted by the National Heart, Lung and Blood Institute (NHLBI) Prevention and Early Treatment of Acute Lung Injury (PETAL) Network. FIRE CORAL will include a subset of participants enrolled in Biology and Longitudinal Epidemiology of PETAL COVID-19 Observational Study (BLUE CORAL), an NHLBI-funded prospective cohort study describing the clinical characteristics, treatments, biology, and outcomes of hospitalized patients with COVID-19 across the PETAL Network. FIRE CORAL consists of a battery of in-person assessments objectively measuring pulmonary function, abnormalities on lung imaging, physical functional status, and biospecimen analyses. Participants will attend and perform initial in-person testing at 3 to 9 months after hospitalization. The primary objective of the study is to determine the feasibility of longitudinal assessments investigating multiple domains of recovery from COVID-19. Secondarily, we will perform descriptive statistics, including the prevalence and characterization of abnormalities on pulmonary function, chest imaging, and functional status. We will also identify potential clinical and biologic factors that predict recovery or the occurrence of persistent impairment of pulmonary function, chest imaging, and functional status.

**Ethics and dissemination:**

FIRE CORAL is approved via the Vanderbilt University central institutional review board (IRB) and via reliance agreement with the site IRBs. Results will be disseminated via the writing group for the protocol committee and reviewed by the PETAL Network publications committee prior to publication. Data obtained via the study will subsequently be made publicly available via NHLBI’s biorepository.

**Strengths and limitations of the study:**

**Strengths:**

- First US-based multicenter cohort of pulmonary and functional outcomes in patients previously hospitalized for COVID-19 infection
- Longitudinal biospecimen measurement allowing for biologic phenotyping of abnormalities
- Geographically diverse cohort allowing for a more generalizable understanding of post-COVID pulmonary sequela

**Limitations:**

- Selected cohort given proximity to a participating center
- Small cohort which may be underpowered to identify small changes in pulmonary function

## Introduction

The long-term consequences of the novel coronavirus SARS-CoV-2 (COVID19) pandemic are yet to be fully realized. Heterogeneous manifestations of disease and syndromes following SARS-CoV-2 infection are common, increasingly recognized, and may be defined in the broadest sense as a failure to return to baseline level of health [1]. Driven in part by unprecedented patient-led advocacy and unprecedented numbers of patients [1–4], there has been increased attention to understanding and treating long-term sequelae of COVID-19, referred to variously as post-acute sequelae of COVID-19 (PASC), post-acute COVID, Long COVID, or long-haul COVID [5]. For example, the National Institutes of Health launched an initiative to advance understanding of PASC in December 2020. The current limited understanding of the diverse long-term sequelae of COVID-19 is a fundamental barrier to improve recovery for patients with PASC.

While the number of investigations focused on patient-reported outcomes is growing, relatively few have reported the results of objective assessments after hospital discharge for patients surviving severe COVID-19 disease [3, 4, 6–8]. Further, most cohorts to date enroll patients with ongoing symptoms rather than the full spectrum of recovery including asymptomatic patients. Despite the high prevalence of COVID-19 infection in the USA, there are limited reports of pulmonary function test (PFT) or computerized tomography (CT) imaging results following hospitalization for COVID-19 with few analyses investigating the relationship between lung function or chest imaging abnormalities and persistent respiratory symptoms. Few US-based cohorts [9] describe integrative functional assessments, such as 6-min walk tests, or longitudinal changes in biomarkers of recovery after hospital discharge.

To address this urgent need for studies that advance understanding of the clinical spectrum of PASC, we describe a protocol for a prospective observational study, the Functional, Imaging, and Respiratory Evaluation (FIRE) of hospitalized patients following COVID-19 (FIRE CORAL), to (1) examine the feasibility of longitudinal assessments investigating multiple domains of recovery in parallel and (2) elucidate the recovery from COVID-19 disease following acute hospitalization with an emphasis on functional, imaging, and respiratory evaluation. The study procedures and assessments chosen by our investigative team for this protocol measure impairments and syndromes commonly described by patients during routine clinical care following COVID-19.

## Methods and analysis

### Study design

FIRE CORAL is a multicenter prospective cohort study of participants recovering from COVID-19 disease with in-person follow-up for functional and pulmonary phenotyping (Table 1). The cohort is comprised of participants who were enrolled and completed study requirements for the National Heart, Lung and Blood Institute (NHLBI) funded BLUE CORAL study (see details below).

**Table 1:**
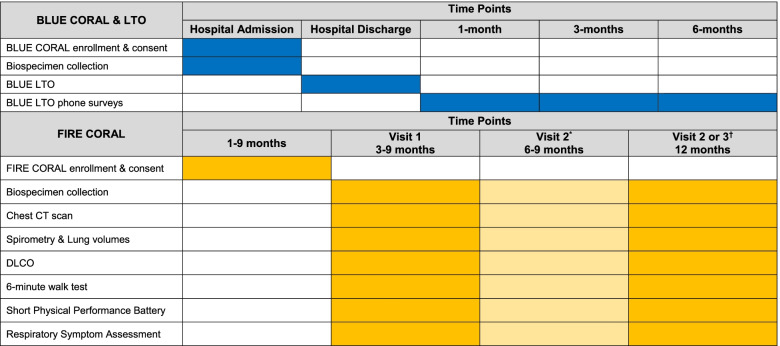
Study procedures for BLUE CORAL, BLUE LTO, and FIRE CORAL

Definitions:

• BLUE CORAL—1390 patient prospective study beginning during acute SARS-CoV2 infection hospitalization

• BLUE LTO—800 patient subset of BLUE CORAL participating in post-hospital telephone follow-up via long-term outcomes team

• FIRE CORAL—80 patient subset of BLUE CORAL LTO, returning for in person assessments

^a^The visit occurring 6–9 months after hospital discharge is optional and only performed if patient completes first FIRE CORAL visit between 3 and 6 months after hospital discharge

bThe visit occurring 12 months after hospital discharge is performed after the previous and at least > 2 months later (window from 11 to 15 months)

Sites from the NHLBI Prevention and Early Treatment of Acute Lung Injury (*PETAL)* Network that are participating in the BLUE CORAL study and have the capabilities of performing study procedures are eligible to participate in the FIRE CORAL study on a volunteer basis. Briefly, PETAL is a research network of 12 Clinical Centers and 1 Clinical Coordinating Center funded by the NHLBI that was initially developed to test prevention and early treatment strategies for patients with or at risk for acute respiratory distress syndrome (ARDS) and during the pandemic expanded to include COVID19 studies including this observational study. Additional information on PETAL network including purpose, coordination, leadership, study oversight, and general study procedures been previously described online (https://petalnet.org/ and https://petalnet.org/studies/public/firecoral).

### Blue coral

BLUE CORAL was a prospective cohort study of up to 1390 hospitalized participants with COVID-19 disease; follow-up of participants is ongoing. The primary aim of BLUE CORAL was to describe the clinical characteristics, treatments, biology, and outcomes of participants hospitalized with COVID-19 infection. Secondarily, BLUE CORAL aimed to identify clinical and biologic risk factors for adverse COVID-19 outcomes and persistent symptoms. These predictors will be used to understand trajectories of recovery and a de-identified repository of biospecimens and clinical data will be established for rapid sharing. Adults (age ≥ 18 years) requiring hospitalization with symptomatic COVID-19 disease were eligible for BLUE CORAL enrollment within 72 h of study hospital admission. Prisoners and patients on hospice/comfort-focused care were excluded. Data on demographics, baseline function, clinical parameters, management, and clinical outcomes as well as biologic specimens were collected during the hospitalization. In addition, participants and/or proxies participate in telephone post-hospital follow-up assessments coordinated between two central call centers at University of Michigan and Oregon Health and Science University at 1, 3, 6, and 12 months after hospital discharge (Table 1). These telephone surveys include assessment of quality of life, ability to participate in activity of daily living including new impairments or disabilities, ongoing symptomology, e.g., dyspnea and fatigue, emotional health symptoms, cognitive function, financial strain/return to employment, and need for rehospitalization.

### FIRE CORAL study aim

The objectives of the FIRE CORAL study are:

1. Primary: To examine the feasibility of conducting rigorous in-person follow-up testing of participants discharged from the hospital following COVID-19 illness to assist with planning for a larger study to evaluate variables associated with differential recovery
2. Secondary: To describe the pulmonary, imaging, and functional recovery following COVID-19 hospitalization in a diverse population of patients. FIRE CORAL extends the BLUE CORAL study follow-up to include in-person study visits for pulmonary phenotyping and functional assessment

### Study setting

**Fig. 1:**
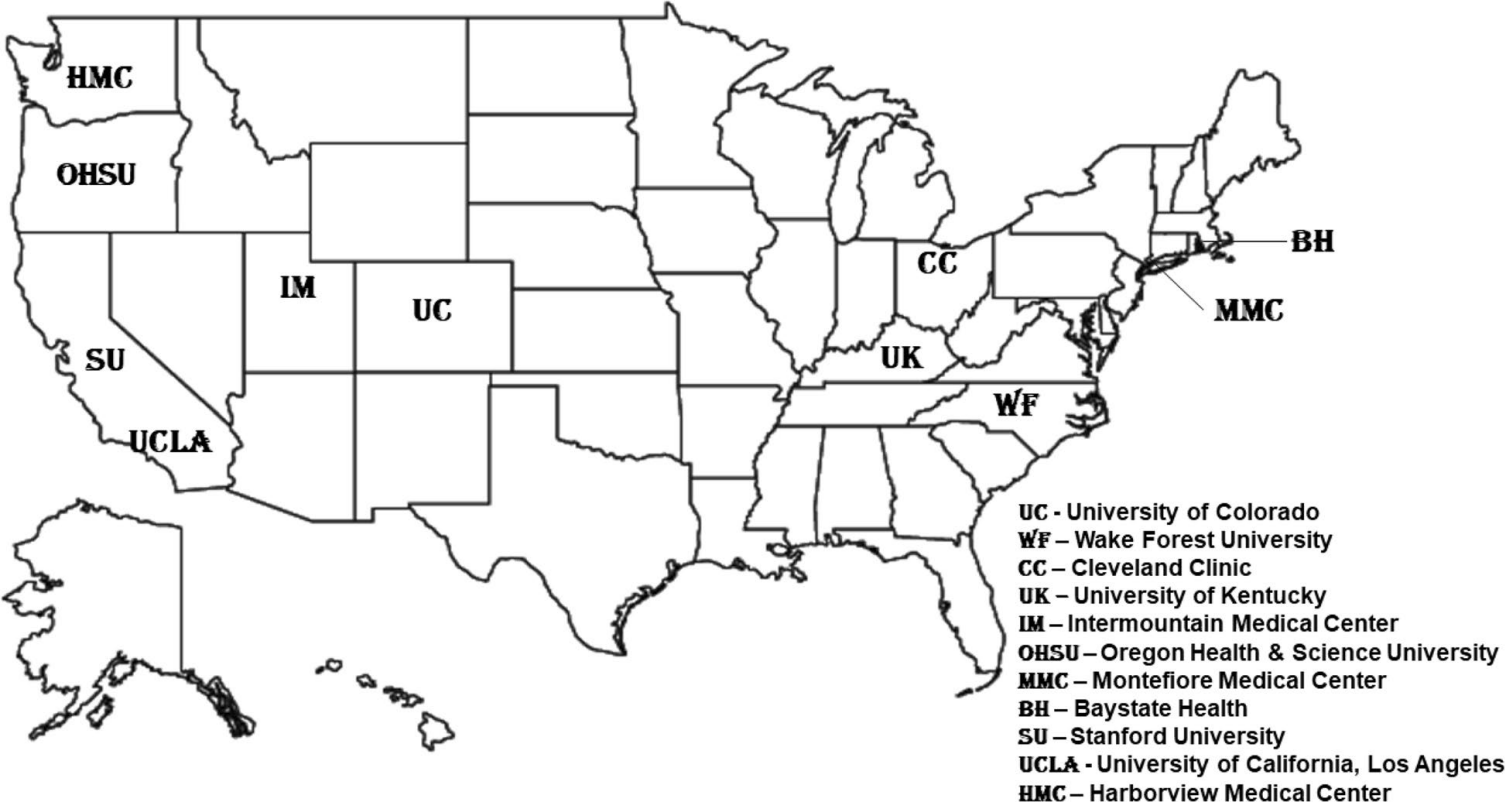
Geographic distribution of participating FIRE-CORAL sites

Ten PETAL Network hospitals geographically spread out across the US are set to participate in FIRE CORAL (Fig. 1). FIRE CORAL will enroll 80 participants from among the cohort of BLUE CORAL participants who survive their hospitalization and are participating in the BLUE CORAL long-term outcomes cohort.

### Inclusion criteria

**Fig. 2:**
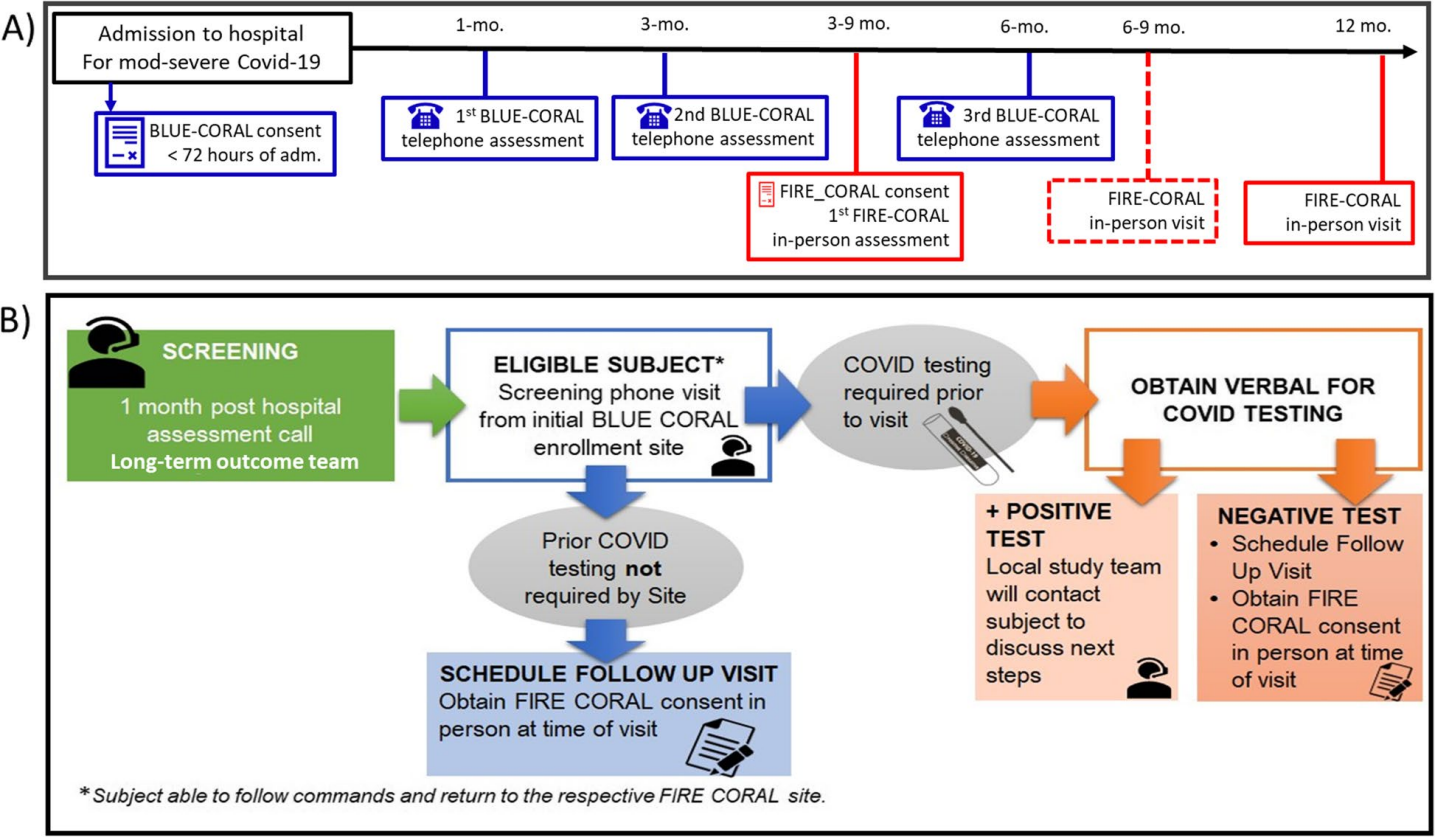
Study inclusion and screening timeline and process. **a** Timeline of eligibility for enrollment in BLUE and FIRE CORAL. **B** Process for site screening and participation based on COVID infection status

We will enroll adult participants with a recent COVID-19 hospitalization who were enrolled in the BLUE CORAL study and completed the 1- or 3-month post-hospital telephone long-term outcomes assessment (Fig. 2A). Participants are eligible for FIRE CORAL if they speak English or Spanish as their primary language and are cleared to attend in-person testing per each local site’s specific COVID-19 infection control criteria. Participants with pre-existing severe disability defined by reduced ADLs and/or cognitive impairment were excluded from post-hospital follow-up and thus not included in FIRE CORAL.

### Exclusion criteria

We will exclude participants unable or unwilling to return to the clinical site for testing, participants unable to follow instructions as reported by caregiver, surrogate or investigator, and participants that self-report pregnancy at time of screening or follow-up visit given the radiation risks associated with CT scans in this population.

### Screening, recruitment, and informed consent

The BLUE CORAL long-term outcome coordinating center will notify FIRE CORAL sites of participants completing the 1- or 3-month telephone assessment for BLUE CORAL who are potentially eligible for participation in FIRE CORAL. Use of a centralized screening center with an empaneled cohort that is already engaged in telephonic follow-up is a unique feature of the FIRE-CORAL cohort (Fig. 2B). This approach aims to improve the likelihood of cohort retention in longer-term post-hospital/ICU follow-up. Once an eligible participant is identified, the participating FIRE-CORAL sites will connect with the eligible participant using a phone script to introduce the study and determine eligibility for inclusion. Those who express interest in participation will be scheduled for an in person visit for study procedures. Informed consent will be obtained from the participant or from a surrogate decision maker if the participant lacks decision-making capacity at the time of the in-person visit.

### Study procedures

**Fig. 3:**
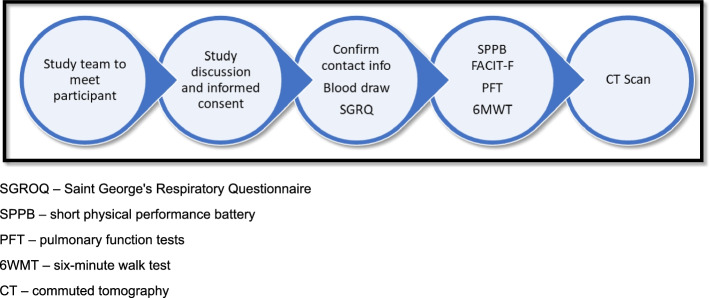
Study day procedures. SGROQ, Saint George’s Respiratory Questionnaire; SPPB, short physical performance battery; PFT, pulmonary function tests; 6WMT, 6-min walk test; CT, commuted tomography

*FIRE CORAL* consists of a battery of in-person assessments objectively measuring pulmonary function, abnormalities on lung imaging, and functional status. Participants will attend and perform initial in-person testing at 3 to 9 months after hospital discharge (Table 1, Fig. 3). Participants with abnormal findings on pulmonary function testing or chest imaging during their initial assessment or those with persistent respiratory symptoms will be eligible to repeat study procedures 3 months later with a subsequent visit at 12 months. All participants will be invited to return for in-person follow-up at 12 months after hospital discharge. Imaging and clinical assessments performed as part of routine clinical care within the specified study timeframe will be used in lieu of dedicated study procedures.

Tests and measures are summarized below:

*A high-resolution chest CT scan* will be performed without contrast to assess residual abnormalities of the lungs according to local clinical protocol. Specifications for site-specific CT protocols were provided by a central radiologist to promote standardization across sites (Table 2) [10]. CT scans will be interpreted by the site radiologist and then subsequently sent via a central repository for standardized reading and scoring by expert chest radiologists at National Jewish Hospital in Denver, CO. These investigators will employ a standard scoring approach developed at National Jewish Hospital for COVID-19 abnormalities as summarized in Table 2. All lung zones will be scored for abnormalities with the extent of involvement quantified to the nearest 10%.

**Table 2:** High-Resolution Chest CT Scoring Criteria developed by National Jewish Hospital, Denver, CO

	RUZ	LUZ	RMZ	LMZ	RLZ	LLZ
Normal attenuation						
**Non-fibrotic abnormality**						
Ground-glass attenuation						
Consolidation						
Ground-glass attenuation with reticular abnormality						
Reticular abnormality						
**Fibrotic abnormality**						
Ground glass abnormality with traction bronchiolectasis/bronchiectasis and/or architectural distortion						
Consolidation with traction bronchiolectasis/bronchiectasis and/or architectural distortion						
Reticular abnormality with traction bronchiolectasis/bronchiectasis and/or architectural distortion						
Honeycombing						
**Other**						
Emphysema						
Cysts/pneumatoceles						
Mosaic attenuation						
Expiratory air trapping						
Pleural effusion: right versus left						
Pneumothorax: right versus left						
Pleural thickening: right versus left						
Mediastinal adenopathy						

Extent of each abnormality is scored in each zone to the nearest 10%, *RUZ* Right upper zone, *LUZ* Left upper zone, *RMZ* Right middle zone, *LMZ* Left middle zone, *RLZ* Right lower zone, *LLZ* Left lower zone

*PFTs* will be performed and will include spirometry, lung volume assessment, and diffusing capacity measured by single breath measurement. These will be collected by the site study teams and reviewed centrally by the research team.

*Six Minute Walk Test (6MWT)* is a valid and reliable measure of functional and exercise capacity in survivors of acute respiratory failure [11]. Participants will undergo the 6MWT per guidelines of the American Thoracic Society [12]. The estimated minimal important difference (MID) for survivors of critical illness ranges between 20 and 30 m, which equates to 3 to 5% of the percent achieved of their calculated predicted distance [11].

*Short Physical Performance Battery (SPPB)* is a valid measure assessing lower-extremity physical function with scores ranging from 0 to 12. The SPPB includes three sub-components: 4-m habitual gait speed, standing balance, and chair-rise test [13, 14].

*St. George’s Respiratory Questionnaire (SGRQ)* is a self-report questionnaire assessing disease-specific health-related quality of life (HRQL) for patients with chronic lung diseases including chronic obstructive pulmonary disease, asthma, and bronchiectasis [15]. The SGRQ scores range from 0 to 100 with a lower score reflecting a better pulmonary-specific HRQL [16–18]. The MID has been proposed between 4 and 8.3 units for patients with COPD [19, 20]. THE SGRQ has been used to describe pulmonary disease-specific HRQL in survivors of ARDS [21].

*Functional Assessment of Chronic Illness Therapy-Fatigue (FACIT-F scale)* is a self-report measure assessing fatigue and the impact upon activities of daily living and function originally developed to examine cancer-related fatigue [22]. The FACIT-F scale has been validated and reliably utilized in patients with COPD [23, 24] and survivors of critical illness [25].

#### Biospecimen collection

Up to 15 mL of blood will be collected, which will include 6 mL of plasma, 2.5 mL for DNA PaxGENE, and 2.5 mL for RNA PaxGENE at each follow-up visit. The overarching goal for these biospecimens is to conduct analyses that advance understanding of the biological responses in participants recovering from severe COVID-19 and that correlate blood measures/biomarkers with clinical phenotypes of poor outcomes. Planned investigations include assessment of longitudinal inflammatory, immunologic or viral response; and evaluation of the association between biologic recovery and long-term symptoms.

#### Demographic and clinical data

Data collected during index hospitalization for the parent BLUE CORAL study will be utilized for participants enrolled in FIRE CORAL. We will abstract data on demographics, COVID testing and symptoms, comorbidities, home and hospital medications, vital signs including ventilator settings, lab values, intensive care interventions, and COVID-19 targeted treatments. All available pre-illness chest imaging, PFTs, and 6MWTs performed within the site’s healthcare system in the 10 years prior to hospital admission for COVID-19 will be collected to establish a pre-COVID-19 baseline for comparison.

#### Study optimization/training

An interdisciplinary protocol development committee was formed in January 2021 with oversight from the PETAL Network and NHLBI. The committee met weekly over the next three months to design the study, including selection and timing of in-person assessments. After the final protocol was approved by protocol committee consensus, the study protocol was reviewed and approved by the PETAL Network Steering Committee, the PETAL Network Data Safety and Monitoring Board, and NHLBI leadership before initiation of primary investigator and research personnel training. All sites across the PETAL Network were invited to participate on a voluntary basis. Personnel involved in the study were required to review the study manuals which included the standard operating procedures. In addition, all personnel attended a video-conference meeting that provided training on each phase of the study including training videos for selected outcome measures. The site initiation webinar and all training videos are readily available on the PETAL Network website.

### Statistical methods

#### Sample size

For this pilot study, the target enrollment was chosen to balance characterizing the range of PASC abnormalities against the time constraints associated with COVID-19 research and the waning pandemic with widespread vaccination. Based on these criteria, the target enrollment of 80 participants was felt to be sufficient to provide an urgently needed, preliminary understanding of longer-term respiratory manifestations after COVID-19 infection sufficient to guide future post-COVID pulmonary studies. Our sample size was purposefully chosen to be small to demonstrate the feasibility of conducting longitudinal in-person research in COVID survivors with an understanding that larger studies would be needed to define disease trajectories and identify clinical predictors with small effects [26]. The sample size of 80 was determined for based on multiple factors including the availability of participants enrolled in BLUE CORAL, the number of participating sites, and previous feasibility studies. Sample sizes for pilot and feasibility studies “should be representative of the target study population,” [27] and in general, there are limited evidence to justify sample size of feasibility studies [26]. Research on feasibility studies recommend sample size estimates based on a confidence interval approach: an expected completion rate of 75% with lower bound of the CI = 70%, a total of 75 participants would be needed [27]. Finally, Sim and Lewis suggest that a sample size of 50 should be utilized for pilot studies of randomized controlled trials (RCT) [28]. Our proposed feasibility study will be deemed successful if at least 50 participants out of proposed 80 (62.5%, 95% CI 52–73%) complete at least one study visit.

#### Statistical analysis plan

To examine the feasibility of long-term follow-up, we will describe the number of participants enrolled and the proportion that returned for in-person follow-up (primary objective). We will perform summary or descriptive statistics to describe the prevalence of abnormalities of pulmonary function, chest CT imaging, and functional status in the enrolled participants (secondary objective). Among participants who return for a 12-month visit, we will describe the trajectory of recovery of pulmonary function and functional status between first and last study visits. Additionally, we will test the relationships (correlative analyses) between outcomes with key independent variables including participant demographics, severity and duration of illness, pre-hospital function, and exposure to in-hospital and post-hospital interventions. Correlative testing and regression analyses will be performed only as *exploratory* to examine relationships between potential predictors and participant outcomes. In addition, repeated measures analyses will be explored to examine change over time. Our small sample size will limit our ability to perform multivariable testing with all potential confounding variables but will allow for identification of potentially important predictors and sample size justification for future larger studies. Our proposed sample is large enough to demonstrate feasibility of longitudinal follow-up, estimate the prevalence of abnormalities after COVID-19 for the purposes of planning, and identify longitudinal trends in recovery and potentially important predictors for future analyses. All analytic plans will be agreed upon by the FIRE CORAL committee after review of the distribution of abnormalities from the first 40 completed visit.

## Data quality, ethics, and dissemination

FIRE CORAL is approved via the PETAL Network central Institutional review board (IRB) at Vanderbilt University and via reliance agreements with the site IRBs. A multi-faceted quality assurance approach will be utilized to ensure data quality and consistency: (1) use of Manuals of Operation for training and reference, (2) regular meetings between local investigators and study coordinators to answer questions and ensure consistency in evaluations across study sites, (3) conferences between all Investigators for the same purposes, (4) ongoing quality assurance review and training updates, (5) data entry into a database with extensive automated checks of data validity, and (6) ongoing review of descriptive statistics by Investigators with detailed review of selected data. The Strengthening of the Reporting of Observational studies in Epidemiology (STROBE) guidelines will be followed for study dissemination [29].

## Discussion

The purpose of the FIRE CORAL study is to comprehensively assess recovery following hospitalization for COVID-19 in a national, multicenter pilot cohort. Data from the study will advance our understanding of the biological, clinical, and patient-related factors associated with long-term impairments following hospitalization for COVID-19 and may inform the development and testing of targeted interventions to mitigate those impairments. Early studies suggest that many patients hospitalized for COVID-19 have persistent symptoms and impairments in respiratory and physical function [6, 30]. Moreover, it is expected that patients requiring an ICU admission for COVID-19 disease may develop impairments associated with post-intensive care syndrome (PICS). Findings from patients surviving ARDS, which is common in individuals hospitalized for COVID-19, demonstrate that long-term impairments lead to reduced quality of life, lost wages with inability to return to work, and increased risk of morbidity, re-hospitalization, and mortality [31–36]. Thus, there is a profound need to understand the recovery of COVID-19 following hospitalization.

FIRE CORAL will expand on the existing literature on COVID recovery in several important and distinct ways. First, FIRE CORAL is unique in that it will enroll participants from geographically and ethnically diverse communities across the USA, increasing the interpretability and generalizability of our findings to the broader pulmonary community. Prior observational cohort studies describing post-COVID impairments following hospitalization were primarily single-center cohort studies from China [37–40] and Europe [41–43]. US cohorts describing post-COVID impairments following hospitalization also remain restricted to regional experiences, and there are no multicenter, national cohorts, to date, describing longitudinal changes in pulmonary function and imaging across the USA [44]. Second, participants in FIRE CORAL will undergo a comprehensive assessment that includes both symptom assessments as well as objective pulmonary and physical functional assessments Most studies to date evaluating patients after COVID-19 hospitalization have used survey methods and focused primarily on patient-reported symptoms [6, 45]. Objective pulmonary measurements, including lung function and CT imaging following COVID-19 hospitalization were reported in some of the observational cohorts earlier in the pandemic from China [37–40] and Europe [41–43]; however, data describing physical recovery following COVID-19 hospitalization remain limited. An additional unique and novel strength of FIRE CORAL will be the availability of biospecimens spanning the recovery trajectory that will help to inform our mechanistic understanding of the development of post-COVID sequela across illness severities. Finally, FIRE CORAL will recruit participants regardless of ongoing symptoms; in fact, some participants will likely have already achieved a “return to baseline health” at their initial post-hospital visit. This may allow us to elucidate a biological pattern of early/sustained recovery in addition to a pattern a persistent inflammation and/or illness.

Methodologically, FIRE CORAL incorporates several innovations with respect to the conduct of longitudinal cohort studies in survivors of serious illness. It leverages existing trial and clinical infrastructure to study longer term outcomes in a more pragmatic, cost-effective manner. Embedding the research infrastructure within existing care models eases the burden of participation for patients, particularly those recovering from severe illness, while also minimizing broader research costs. Additionally, this allows for direct, real-time feedback on care delivery for COVID19 survivors reducing the implementation gap that exists between research and clinical care. Therefore, even as a pilot study, FIRE CORAL has the potential to inform current practices in research on long-term functional outcomes of acute respiratory illness by demonstrating the feasibility of incorporating research activities across the care continuum for hospital survivors and building a network of facilities equipped for trials in the post-hospital setting. Inclusion of a geographically diverse network of US institutions provides a more generalizable understanding of the post-hospital COVID-19 experience and allows for targeted enrollment in centers with larger proportions of underserved and underrepresented communities to better understand disparities in post-COVID recovery. Together, these features will make FIRE CORAL an important contribution to the burgeoning literature on PASC.

## Conclusions

The enclosed FIRE CORAL protocol presents a novel, longitudinal cohort of post-COVID-19 pulmonary and functional recovery after hospital discharge. This in person phenotyping will extend our knowledge of the long-term effects of COVID-19 and provide insight into biologic mechanisms of incomplete pulmonary recovery after COVID-19. This will establish a longer-term biorepository after COVID-19 that will help to inform future studies of COVID-19 recovery.

## Data Availability

Not applicable.

## Abbreviations

ARDS: Acute respiratory distress syndrome
BLUE CORAL: Biology and Longitudinal Epidemiology of PETAL COVID-19 Observational Study
COVID19: Novel coronavirus SARS-Cov2
CT scan: Computerized tomography scan
FIRE: Functional, Imaging, and Respiratory Evaluation
HRQL: Health-related Quality of Life
IRB: Institutional Review Board
LTO: Long-term outcomes
NHLBI: National Heart, Lung and Blood Institute
PASC: Post-Acute Sequela of COVID19
PETAL Network: Prevention and Early Treatment of Acute Lung Injury Network
PFT: Pulmonary function test

## References

[1] Lerner AM, Robinson DA, Yang L, Williams CF, Newman LM, Breen JJ, et al. Toward Understanding COVID-19 Recovery: National Institutes of Health Workshop on Postacute COVID-19. Ann Intern Med. 2021;174(7):999–1003. 10.7326/M21-1043. 10.7326/M21-1043 PMC8025940 33780290

[2] Zhou J, Liu B, Liang C, Li Y, Song YH (2016). Cytokine signaling in skeletal muscle wasting. Trends Endocrinol Metab.

[3] Ghosn J, Piroth L, Epaulard O, Le Turnier P, Mentré F, Bachelet D, Laouénan C. French COVID cohort study and investigators groups. Persistent COVID-19 symptoms are highly prevalent 6 months after hospitalization: results from a large prospective cohort. Clin Microbiol Infect. 2021;27(7):1041.e1–1041.e4. 10.1016/j.cmi.2021.03.012. 10.1016/j.cmi.2021.03.012 PMC8107834 34125067

[4] Huang C, Huang L, Wang Y (2021). 6-month consequences of COVID-19 in patients discharged from hospital: a cohort study. Lancet.

[5] Rubin R (2020). As Their Numbers Grow, COVID-19 “Long Haulers” stump experts. JAMA.

[6] Carfì A, Bernabei R, Landi F, Group ftGAC-P-ACS (2020). Persistent Symptoms in patients after acute COVID-19. JAMA.

[7] Self WH, Tenforde MW, Stubblefield WB (2020). Seroprevalence of SARS-CoV-2 Among frontline health care personnel in a multistate hospital network - 13 academic medical centers, April-June 2020. MMWR Morb Mortal Wkly Rep.

[8] Halpin S, O'Connor R, Sivan M (2021). Long COVID and chronic COVID syndromes. J Med Virol.

[9] McGroder CF, Zhang D, Choudhury MA, Salvatore MM, D'Souza BM, Hoffman EA, et al. Pulmonary fibrosis 4 months after COVID-19 is associated with severity of illness and blood leucocyte telomere length. Thorax. 2021;76(12):1242–5. 10.1136/thoraxjnl-2021-217031. 10.1136/thoraxjnl-2021-217031 PMC8103561 33927016

[10] Burnham EL, Hyzy RC, Paine R (2013). Chest CT features are associated with poorer quality of life in acute lung injury survivors. Crit Care Med.

[11] Chan KS, Pfoh ER, Denehy L (2015). Construct validity and minimal important difference of 6-minute walk distance in survivors of acute respiratory failure. Chest.

[12] ATS Committee on Proficiency Standards for Clinical Pulmonary Function Laboratories. ATS statement: guidelines for the six-minute walk test. Am J Respir Crit Care Med. 2002;166(1):111–7. 10.1164/ajrccm.166.1.at1102. Erratum in: Am J Respir Crit Care Med. 2016;193(10):1185. PMID:12091180. 10.1164/ajrccm.166.1.at1102 12091180

[13] Chan KS, Aronson Friedman L, Dinglas VD (2016). Evaluating physical outcomes in acute respiratory distress syndrome survivors: validity, responsiveness, and minimal important difference of 4-meter gait speed test. Crit Care Med.

[14] Parry SM, Denehy L, Beach LJ, Berney S, Williamson HC, Granger CL (2015). Functional outcomes in ICU - what should we be using? - an observational study. Crit Care.

[15] Jones PW, Quirk FH, Baveystock CM (1991). The St George's Respiratory Questionnaire. Respir Med.

[16] Wilson CB, Jones PW, O'Leary CJ, Cole PJ, Wilson R (1997). Validation of the St. George's Respiratory Questionnaire in bronchiectasis. Am J respiratory Crit Care Med.

[17] Jones PW (1994). Quality of life, symptoms and pulmonary function in asthma: long-term treatment with nedocromil sodium examined in a controlled multicentre trial. Nedocromil Sodium Quality of Life Study Group. Eur Respir J.

[18] Jones PW, Quirk FH, Baveystock CM, Littlejohns P (1992). A self-complete measure of health status for chronic airflow limitation. The. St George's Respiratory Questionnaire. Am Rev Respir Dis.

[19] Jones PW (2005). St. George's Respiratory Questionnaire: MCID. COPD.

[20] Welling JBA, Hartman JE, Ten Hacken NHT, Klooster K, Slebos D-J (2015). The minimal important difference for the St George's Respiratory Questionnaire in patients with severe COPD. Eur Respir J.

[21] Davidson TA, Caldwell ES, Curtis JR, Hudson LD, Steinberg KP (1999). Reduced quality of life in survivors of acute respiratory distress syndrome compared with critically ill control patients. JAMA.

[22] Yellen SB, Cella DF, Webster K, Blendowski C, Kaplan E (1997). Measuring fatigue and other anemia-related symptoms with the Functional Assessment of Cancer Therapy (FACT) measurement system. J Pain Symptom Manage.

[23] Al-shair K, Muellerova H, Yorke J (2012). Examining fatigue in COPD: development, validity and reliability of a modified version of FACIT-F scale. Health Qual Life Outcomes.

[24] Knorst MM, Coertjens PC, Coertjens M, Coelho AC (2019). Factors associated with fatigue in chronic obstructive pulmonary disease: a cross-sectional study. European Respiratory Journal.

[25] Spadaro S, Capuzzo M, Valpiani G (2016). Fatigue in intensive care survivors one year after discharge. Health Qual Life Outcomes.

[26] Billingham SA, Whitehead AL, Julious SA (2013). An audit of sample sizes for pilot and feasibility trials being undertaken in the United Kingdom registered in the United Kingdom Clinical Research Network database. BMC Med Res Methodol.

[27] Thabane L, Ma J, Chu R (2010). A tutorial on pilot studies: the what, why and how. BMC Med Res Methodol.

[28] Sim J, Lewis M (2012). The size of a pilot study for a clinical trial should be calculated in relation to considerations of precision and efficiency. J Clin Epidemiol.

[29] Elm Ev, Altman DG, Egger M, Pocock SJ, Gøtzsche PC, Vandenbroucke JP (2007). Strengthening the reporting of observational studies in epidemiology (STROBE) statement: guidelines for reporting observational studies. BMJ.

[30] Chopra V, Flanders SA, O'Malley M, Malani AN, Prescott HC (2021). Sixty-day outcomes among patients hospitalized with COVID-19. Ann Intern Med.

[31] Biehl M, Kashyap R, Ahmed AH (2015). Six-month quality-of-life and functional status of acute respiratory distress syndrome survivors compared to patients at risk: a population-based study. Critical care (London, England).

[32] Dinglas VD, Aronson Friedman L, Colantuoni E (2017). Muscle weakness and 5-year survival in acute respiratory distress syndrome survivors. Crit Care Med.

[33] Kamdar BB, Huang M, Dinglas VD (2017). Joblessness and lost earnings after acute respiratory distress syndrome in a 1-year national multicenter study. Am J Respir Crit Care Med.

[34] Marti J, Hall P, Hamilton P (2016). One-year resource utilisation, costs and quality of life in patients with acute respiratory distress syndrome (ARDS): secondary analysis of a randomised controlled trial. J Intensive Care.

[35] Bienvenu OJ, Colantuoni E, Mendez-Tellez PA (2015). Cooccurrence of and remission from general anxiety, depression, and posttraumatic stress disorder symptoms after acute lung injury: a 2-year longitudinal study. Crit Care Med.

[36] Herridge MS, Moss M, Hough CL (2016). Recovery and outcomes after the acute respiratory distress syndrome (ARDS) in patients and their family caregivers. Intensive Care Med.

[37] Zhao YM, Shang YM, Song WB (2020). Follow-up study of the pulmonary function and related physiological characteristics of COVID-19 survivors three months after recovery. EClinicalMedicine.

[38] Qin W, Chen S, Zhang Y, Dong F, Zhang Z, Hu B, et al. Diffusion capacity abnormalities for carbon monoxide in patients with COVID-19 at 3-month follow-up. Eur Respir J. 2021;58(1):2003677. 10.1183/13993003.03677-2020. 10.1183/13993003.03677-2020 PMC7877322 33574077

[39] Wu X, Liu X, Zhou Y (2021). 3-month, 6-month, 9-month, and 12-month respiratory outcomes in patients following COVID-19-related hospitalisation: a prospective study. Lancet Respir Med.

[40] Huang C, Huang L, Wang Y (2021). 6-month consequences of COVID-19 in patients discharged from hospital: a cohort study. Lancet (London, England).

[41] Morin L, Savale L, Pham T (2021). Four-month clinical status of a cohort of patients after hospitalization for COVID-19. JAMA.

[42] Frija-Masson J, Debray MP, Boussouar S (2021). Residual ground glass opacities three months after COVID-19 pneumonia correlate to alteration of respiratory function: the post COVID M3 study. Respir Med.

[43] Balbi M, Conti C, Imeri G (2021). Post-discharge chest CT findings and pulmonary function tests in severe COVID-19 patients. Eur J Radiol.

[44] Taquet M, Dercon Q, Luciano S, Geddes JR, Husain M, Harrison PJ (2021). Incidence, co-occurrence, and evolution of long-COVID features: a 6-month retrospective cohort study of 273,618 survivors of COVID-19. PLoS Med.

[45] Havervall S, Rosell A, Phillipson M (2021). Symptoms and functional impairment assessed 8 months after mild COVID-19 among health care workers. JAMA.

